# Identification of Significant Genes in Lung Cancer of Nonsmoking Women via Bioinformatics Analysis

**DOI:** 10.1155/2021/5516218

**Published:** 2021-10-11

**Authors:** Yu Wang, Sibo Hu, Xianguang Bai, Ke Zhang, Ruixue Yu, Xichao Xia, Xinhua Zheng

**Affiliations:** ^1^College of Medicine, Pingdingshan University, Pingdingshan, Henan, China; ^2^Department of Physical Education, Henan University of Urban Construction, Pingdingshan, Henan, China

## Abstract

**Background:**

The aim of this study was to identify potential key genes, proteins, and associated interaction networks for the development of lung cancer in nonsmoking women through a bioinformatics approach.

**Methods:**

We used the GSE19804 dataset, which includes 60 lung cancer and corresponding paracancerous tissue samples from nonsmoking women, to perform the work. The GSE19804 microarray was downloaded from the GEO database and differentially expressed genes were identified using the limma package analysis in R software, with the screening criteria of *p* value < 0.01 and ∣log_2_ fold change (FC) | >2.

**Results:**

A total of 169 DEGs including 130 upregulated genes and 39 downregulated were selected. Gene Ontology and KEGG pathway analysis were performed using the DAVID website, and protein-protein interaction (PPI) networks were constructed and the hub gene module was screened through STING and Cytoscape.

**Conclusions:**

We obtained five key genes such as GREM1, MMP11, SPP1, FOSB, and IL33 which were strongly associated with lung cancer in nonsmoking women, which improved understanding and could serve as new therapeutic targets, but their functionality needs further experimental verification.

## 1. Introduction

Until recently, lung cancer (LC) is the malignant neoplasm with the highest incidence and mortality worldwide, the tumor with the highest cancer mortality rate among men and the second highest cancer mortality rate among women [1]. Smoking is the major independent risk factor in the development of LC [2, 3]. Nonetheless, 15% of men and 53% of women with LC have never consumed tobacco [4]. Among them, nonsmokers are more common in women with lung cancer [5]. Therefore, there might be many other important factors that affect the occurrence and development of lung cancer in nonsmokers, such as air pollution, second-hand smoke, genetic factors, and occupational exposure. Although many genes have been screened to understand the causes of lung cancer in nonsmoking female patients, such as TP53 [6], PI3K [7], EML4-ALK [8], and BIRC5 [9], the molecular mechanism is still unclear. It is crucial to recognize the unique molecular phenotypic characteristics of nonsmokers with lung cancer for early diagnosis and targeted therapy.

With the development of gene microarray technology and the application of bioinformatics tools, whole gene expression profiling can be used to compare the expression changes of thousands of genes simultaneously and comprehensively screen all relevant genes of cancer, as well as to reveal the interrelationship between different gene expression changes, thus providing clues for studying the intrinsic connection between genes [10–12]. Bioinformatics-based data analysis plays an important role in the study of oncology [13]. In recent years, a large number of gene microarray datasets have been developed from lung cancer specimens, from which a series of differentially expressed genes (DEGs) have been identified, and gene annotation and pathway functions have been carried out [14, 15]. The analysis of DEGs may provide a possibility for diagnosis marker and therapeutic targets at the molecular level of LC.

**Figure 1:**
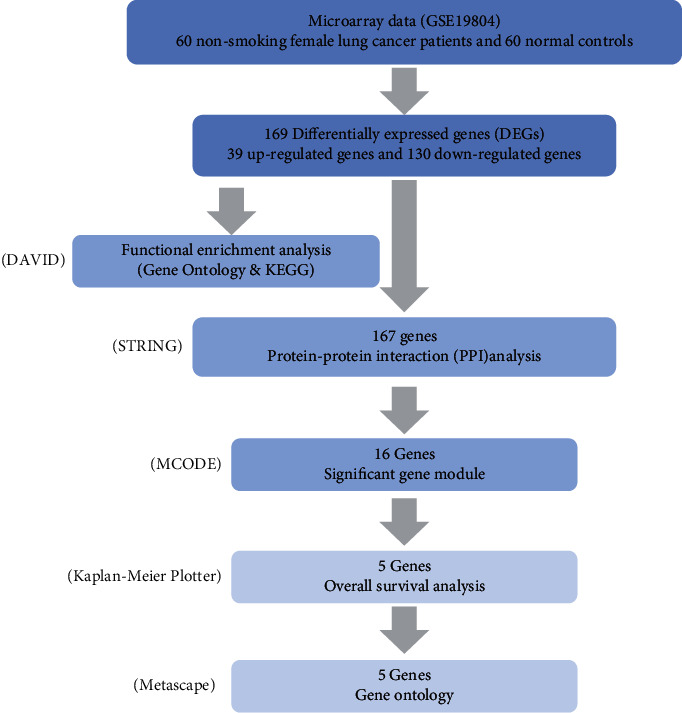
The frame of this study.

In this work, we acquired mRNA expression profiles from the GSE19804 dataset through the GEO website (https://www.ncbi.nlm.nih.gov/geo/), which is a public database that allows archiving, uploading, and querying microarrays. A total of 60 nonsmoking women with lung cancer were absorbed in GSE19804 [16, 17], and samples were collected from tumor (marked cancer) and adjacent normal tissue (marked normal). We used the limma [18] package built in R [19] software to obtain DEGs from mRNA expression profiling data and categorized them into up- and downregulated genes. Then, gene function and pathway analysis of DEGs was performed with DAVID (https://david.ncifcrf.gov/). Protein-protein interaction (PPI) network was conducted by STRING [20] (https://string-db.org/) and visualized by Cytoscape [21]. The core gene module (Module 1) of the network was identified by MCODE [22] app. Ultimately, we performed overall survival analysis for each gene in Module 1 via the Kaplan-Meier Plotter [23] (https://kmplot.com/analysis/). By applying this approach, the genes that were flitted may be associated with the development of lung cancer in nonsmoking women, which were identified as potential biomarkers for diagnosis, prognosis, therapeutic targets, and clinical pharmaceutical research. The framework of this study is shown in Figure 1.

## 2. Methods and Materials

### 2.1. Data Acquisition and Preprocessing

The microarray data GSE19804 [16, 17] was downloaded from the Gene Expression Omnibus (GEO) database (https://www.ncbi.nlm.nih.gov/geo/). The platform of GSE19804 was Affymetrix Human Genome U133 Plus 2.0 Array, which contained 60 female lung cancer patients who have never smoked and 60 normal controls. mRNA expression matrices of patients can be obtained from the microarray. All the samples were collected from Taiwan. Subsequently, the probe identification numbers were transformed into official gene symbols. After deletion of duplicate genes, one-to-many and non-mRNA probes, the next differential gene analysis was performed on all gene expression data.

### 2.2. Identification of DEGs

DEG analysis is the finding of statistically significant genes from the multitude of genetic information on gene expression microarrays. All the gene expression data were analyzed using the limma package of R Studio [19, 24] software. The limma [18] package performs differential analysis of gene expression data and experimental design through linear modeling. We applied the limma package for preliminary DEG screening of tumor tissues (marked cancer) and adjacent normal tissues (marked normal). With the parameters of the filter set to ∣log_2_ fold change (FC) | ≥2 and adjust *p* value < 0.01, the resulting DEGs would proceed to the next step.

### 2.3. Functional and Signal Pathway Enrichment Analysis

Gene Ontology (GO) [25] is the standardized portrayal or semantic interpretation of terms used to characterize genes and their products, including biological process (BP), cellular component (CC), and MF (molecular function). The KEGG pathway [26] is a set of manually drawn pathway maps representing our understanding of molecular interactions, reactions, and networks of relationships in metabolism, genetic information processing, etc. We used the online tool DAVID [27] website (https://david.ncifcrf.gov/) for GO and KEGG pathway annotation of candidate genes, setting the terms to “Homo sapiens,” *p* value < 0.05.

### 2.4. Protein-Protein Interaction Analysis and Gene Module Analysis

To understand the interactions between DEGs, PPI queries were conducted. PPI analysis was carried out using STRING [20, 28] (https://string-db.org/, version 11.0) database, which is an online tool to search known protein interactions. We uploaded DEGs to the system and set the minimum required interaction score > 0.150 as restriction. The active interaction sources included text mining, experiments, databases, coexpression, neighborhood, gene fusion, and cooccurrence. The results from the PPI analysis were visualized by applying the Cytoscape [21] software (version 3.7.2). Subsequently, an app named MCODE [22] (https://apps.cytoscape.org/apps/mcode, version 1.6.1) was utilized to find the core module in the networks. The MCODE parameters were set to the default value, except *K*‐core = 6.

### 2.5. Overall Survival Analysis Validation and Gene Ontology

To verify whether hub gene expression has survival significance in nonsmoking female lung cancer patients, overall survival analysis was performed. The genes in the previous module were verified for overall survival analysis with the Kaplan-Meier Plotter [23] (https://kmplot.com/analysis/). This online tool integrated the gene expression and clinical data from GEO, EGA, and TCGA, including lung cancer (*n* = 3452), which can evaluate the impact of 54k genes (mRNA, miRNA, and protein) on cancer survival. The Kaplan-Meier survival chart was conducted to compare the two patient cohorts, and the 95% confidence interval and logrank value hazard ratio were calculated. We selected the types of diseases as “lung cancer,” setting limits as follows: “gender: female,” “smoking history: only those never smoked,” “split patients by the following: lower tertile” and “logrank value < 0.05.” Survival was evaluated using the Kaplan-Meier survival curves. Ultimately, the genes verified by survival analysis were subjected to GO analysis using Metascape [29] (https://metascape.org/gp/index.html).

## 3. Results

### 3.1. Identification of DEGs

**Figure 2:**
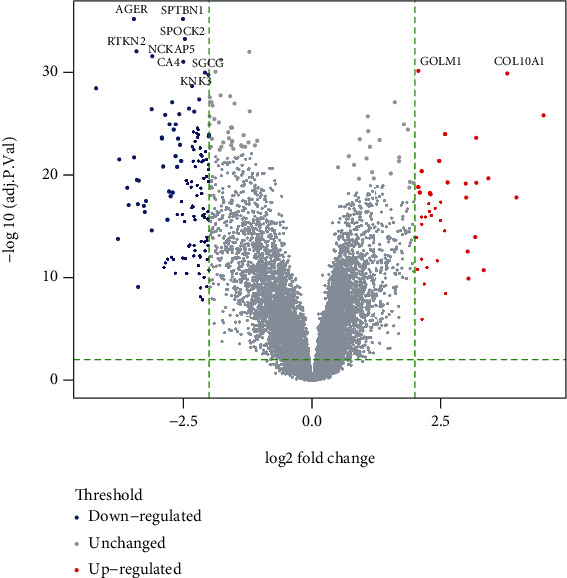
The volcano plot of DEGs. Volcano plot of 139 differentially expressed genes (DEGs). Blue indicates downregulated genes, and red indicates upregulated genes. The top 10 differentially expressed genes have been marked in the figure.

By analyzing the gene expression microarrays GSE19804, with criteria as ∣log_2_ FC | ≥2 and adjust *p* value < 0.01, a total of 169 DEGs were selected, including 39 upregulated genes and 130 downregulated genes (Figure 2 and Table 1). The top 10 upregulated genes were SPP1, COL11A1, COL10A1, HS6ST2, SPINK1, TOX3, CTHRC1, MMP12, MMP1, and GREM1. The top 10 downregulated genes were MMRN1, KCNK3, RXFP1, RAMP3, SOCS2, FOXF1, FIBIN, KANK3, HBEGF, and PCOLCE2.

### 3.2. Functional and Signal Pathway Enrichment Analysis

**Figure 3:**
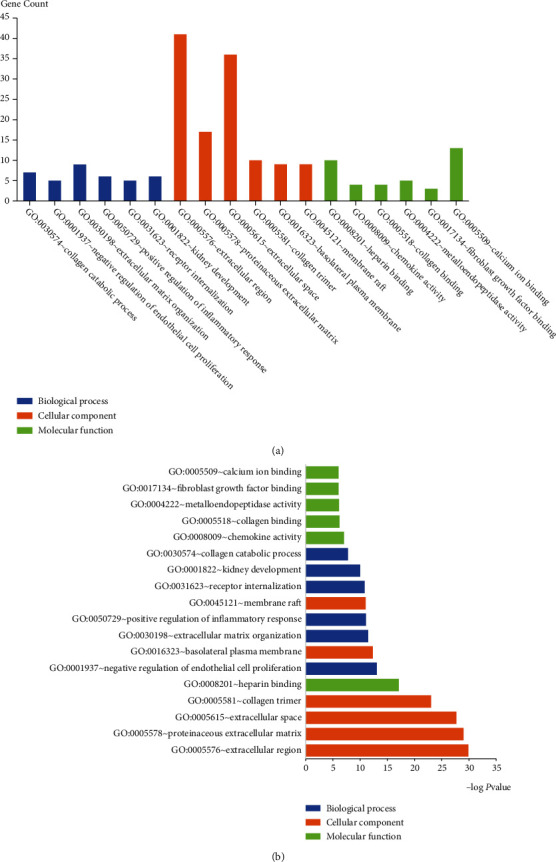
The Gene Ontology and KEGG pathway of DEGs. (a) The top 6 functional enrichment analysis of DEGs in biological process (BP), cellular component (CC), and molecular function (MF). (b) The top 6 functional enrichment analysis arranged according to adj. *p* value.

**Figure 4:**
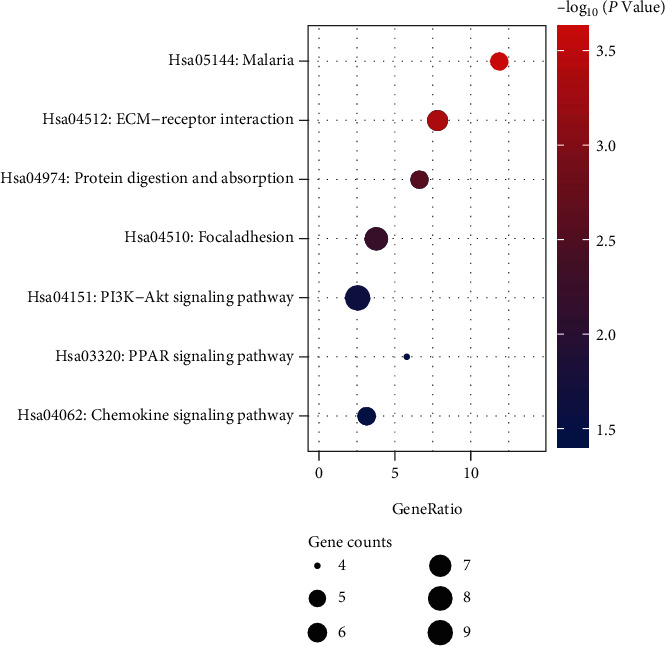
The KEGG pathway of DEGs.

By performing GO and KEGG pathway analysis of DEGs through the DAVID website, we classified them into three terms: biological process (BP), cellular component (CC), and molecular function (MF). It can be seen from Table 2 and Figure 3. The top 3 significant distributions of BP enrichment were “collagen catabolism,” “negative regulation of endothelial cell proliferation,” and “extracellular matrix tissue”; CC were “extracellular area,” “protein extracellular matrix,” and “extracellular space”; and MF were “heparin binding,” “chemokine activity,” and “collagen binding,” respectively. Through the analysis of the KEGG pathway on the DAVID website, the DEGs were enriched in the following 7 pathways (Figure 4 and Table 3). The top 3 pathways with the largest differences were “malaria,” “ECM-receptor interaction,” and “protein digestion and absorption.”

### 3.3. Protein-Protein Interaction Analysis and Gene Module Analysis

**Figure 5:**
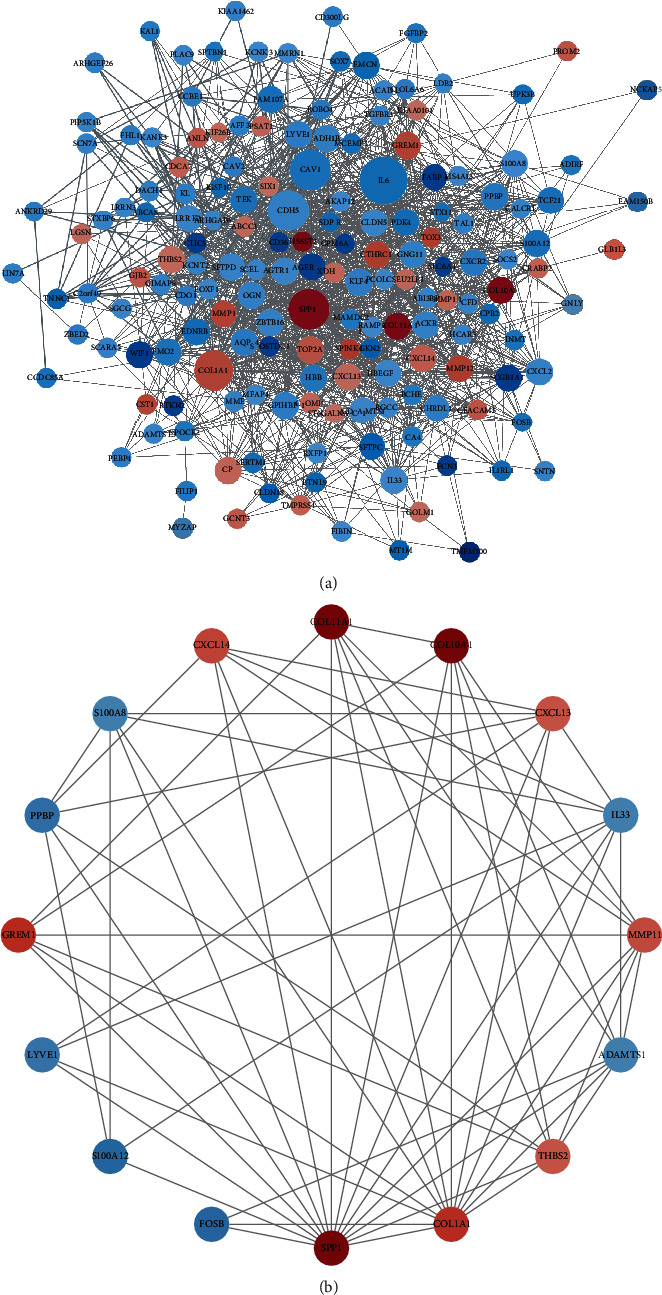
The protein-to-protein interaction network. (a) The overview of the PPI network, with 167 nodes/genes and 1357 edges, including 129 downregulated (marked blue) and 38 upregulated (marked red) genes. The color shade of the nodes was set according to log FC *p* value of DEGs, and the size was set according to the edges. (b) Module 1 consisted of 16 nodes/genes.

We uploaded 169 DEGs to the STRING website and deleted the disconnected nodes to form a network of 167 nodes/genes and 1357 edges, including 129 downregulated and 38 upregulated genes. Then, the network was imported into the Cytoscape software for visualization (Figure 5(a)). Moreover, based on MCODE, with *K*‐core = 6 as criteria, the most significant gene module (Module 1) was selected, which contained 16 node/genes and 58 edges (Figure 5(b)). In Module 1, 9 genes were upregulated (COL10A1, COL11A1, COL1A1, CXCL13, CXCL14, GREM1, MMP11, SPP1, and THBS2) and 7 genes were downregulated (FOSB, IL33, LYVE1, PPBP, S100A12, and S100A8). The 16 genes above would be selected for the next step verification.

### 3.4. Overall Survival Analysis Validation and Gene Ontology

**Figure 6:**
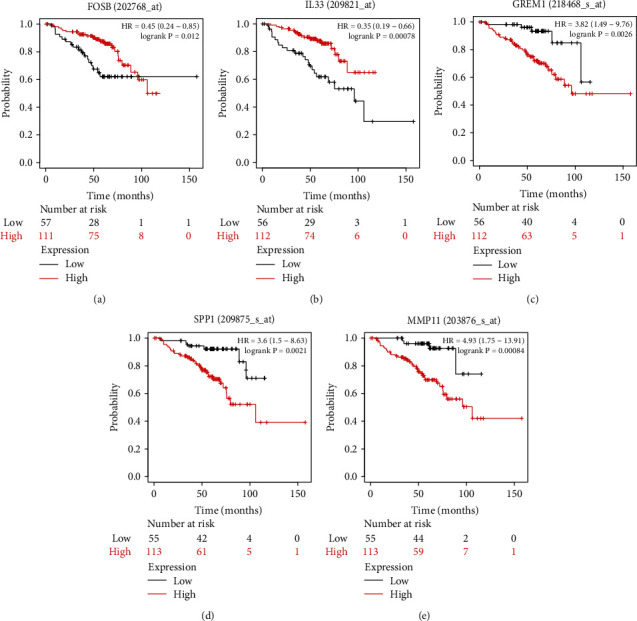
The overall survival of the 5 genes in female LC of nonsmokers. Downregulated genes ((a) FOSB, (b) IL33) and upregulated genes ((c) GREM1, (d) MMP11, and (e) SPP1).

In order to evaluate the clinical significance of these genes in nonsmoking female lung cancer patients, we imported the 16 candidate genes into the Kaplan-Meier Plotter for overall survival analysis verification. Using the selected parameters, the analysis runs on 168 patients. As shown in Figure 6, there were 5 genes that meet the screening requirements. The upregulated genes of GREM1 (HR = 3.82) (Figure 6(c)), SPP1 (HR = 3.6) (Figure 6(d)), and MMP11 (HR = 4.93) (Figure 6(e)) had lower survival rate in the high expression group compared with the low expression group, while the downregulated genes of FOSB (HR = 0.45) (Figure 6(a)) and IL33 (HR = 0.35) (Figure 6(b)) were the opposite. Gene Ontology of 5 hub genes was performed through Metascape (Table 4).

## 4. Discussion

Gene microarray technology is one of the most important methods for exploring gene expression and is particularly relevant in the study of complex refractory diseases [30]. It is well known that smoking is the most important independent risk factor for LC, but a proportion of lung cancer patients have never smoked, which is more frequent among females. As previously described, nonsmoking lung cancer could be classified as a unique type according to the unique genome and molecular mechanism [31]. Although many genes, such as CYP1A1 [32], ERCC2 [33], and L10 [34], have been confirmed to relate to nonsmoking lung cancer, the mechanism related to nonsmoking female lung cancer patients is not clear. The purposes of our study were to explore novel potential genes through comparing 60 LC women tissue without tobacco consumption with the adjacent normal tissue. By using R software, we identified 39 upregulated and 130 downregulated DEGs from GSE19804 downloaded from the GEO database. Following GO and KEGG pathway analysis, PPI network of DEGs was performed and the most significant gene module (Module 1) was selected, from which 16 genes were chosen to validate overall survival in the Kaplan-Meier Plotter. Finally, a total of 5 genes, GREM1, MMP11, SPP1, FOSB, and IL33, were screened out as potential biological markers.

We implemented GO and KEGG pathway analysis using the DAVID online tool to identify BP, CC, and MF and pathways involved in DEGs. With regard to BP, DEGs are mainly enriched in collagen catabolic process, extracellular matrix organization, and positive regulation of inflammatory response. In fact, collagen metabolism and extracellular matrix organization are widely involved in the growth, metastasis [35], and immunosuppression [36] of lung cancer. The genes identified of this study have been shown to be associated the promotion of collagen metabolism in tumors, with mmp11 leading to LC progression through regulation of collagen catabolism and fibrous tissue. DEGs in CC are majorly enriched in extracellular region, proteinaceous extracellular matrix, and collagen trimer. In consonance with this, DEGs are predominantly associated with collagen binding and chemokine and metalloendopeptidase activity in MF. Collagen provides a scaffold for extracellular matrix (ECM) assembly and promotes cancer cell migration and invasion [37]. It has also been reported that intratumoral collagen is a major source of immunosuppression and resistance to PD-1/PD-L1 axis blockade [37]. The genes identified in this study have been shown to be involved in the promotion of collagen metabolism in LC, with MMP11 leading to LC progression through regulation of collagen catabolism and fibrous tissue [38].

Next, interrelationship analysis of the pathway was performed using the KEGG process in DAVID. DEGs were mainly associated with ECM-receptor interaction including COL11A1, COL1A1, THBS2, and SPP1. The ECM can be classified into interstitial matrix (IM) and basement membrane (BM), in which renewal and degradation are intrinsically linked to the invasive phenotype of malignant cells [39]. Furthermore, COL11A1, encoding collagen type XI *α*1, was overexpressed in recurrent and metastatic NSCLC and promotes proliferation, invasion, and migration of NSCLC via the Smad signaling pathway [40]. Additionally, SPP1 (osteopontin) was enriched in both the ECM-receptor interaction and PI3K-Akt signaling pathway. SPP1 is an important component of ECM, regulating matrix interactions and cell adhesion [41]. SPP1 promotes tumorigenesis and metastasis through accumulation of vascular endothelial growth factor (VEGF) [42] and facilitates immune escape from tumors through upregulation of PD-L1 tumor-associated macrophages [43]. Also, fibroblasts differentiated from bone marrow CD4+ monocytes enhance the cancer hepatocyte-like properties of LC cells through the secretion of SPP1 and activation of the PIK3K/AKT pathway [44]. We also found that PIK3K/AKT was a significantly enriched pathway. Activation of the PI3K/AKT pathway may lead to upregulation of tumors via VEGF, resulting in tumors with angiogenic properties [45]. In consensus, both SPP1 and COL11A1 were found as upregulated DEGs in this work, indicating that the ECM-receptor interaction and PIK3K/AKT signaling pathway might play a key role in nonsmoking female patients of LC.

Although the GSE19804 datasets have been mined several times, our work focused on gene expression differences in lung cancer in nonsmoking women and therefore has certain uniqueness in terms of data mining perspectives, methods, and results compared to existing studies. Firstly, the perspective of analysis is different, as the main way of using these datasets was to study the gene expression differences between non-small-cell lung cancer (NSCLC) and normal tissues, without using smoking as a qualifying study condition [46–49]. Secondly, many studies target the significance of single gene expression in NSCLC, especially oncogenes, such as cyclin B2 (CCNB2) [50], pituitary tumor transforming gene-1 (PTTG1) [51], and tumor suppressor gene as hedgehog-interacting protein (HHIP) [52]. Of course, there are also studies on nonsmoking lung cancer in women. In screening for DEGs, we used the limma package in R software, but Yang et al. [9] used the GEO2R (https://www.ncbi.nlm.nih.gov/geo/geo2r/), which is an online tool of the GEO database. So we screened for different DEGs and pathways. In addition, some studies are screening for miRNA [53] and lncRNA [54].

## 5. Conclusion

This study investigated the potential candidate genes and signaling pathways of DEGs in lung cancer with nonsmoking women by analyzing the GSE19804 microarrays. Genes were selected by DEG, GO, KEGG, and PPI analysis. Finally, the upregulated (GREM1, MMP11, and SPP1) and downregulated (FOSB, IL33) genes were screened. This study improves our understanding of the pathogenesis and underlying molecular mechanisms of lung cancer in nonsmoking women. These selected candidate genes and pathways could give us a clue for a new therapeutic target. However, determining the function of these molecules requires further molecular biology experimental validation.

## Figures and Tables

**Table 1:** The differentially expressed genes (DEGs). 169 DEGs were identified from GSE19804, with 39 upregulated and 130 downregulated genes expressed in nonsmoking female lung cancer patients compared to adjacent normal controls. Each group was sorted by fold change, from the largest to smallest (∣log_2_ FC | ≥2, adjust *p* value < 0.01).

DEGs	Gene name
Upregulated	SPP1, COL11A1, COL10A1, HS6ST2, SPINK1, TOX3, CTHRC1, MMP12, MMP1, GREM1, COL1A1, CST1, TOP2A, CEACAM5, PROM2, GJB2, AFAP1-AS1, ANLN, GCNT3, CXCL14, CDCA7, TMPRSS4, CRABP2, PSAT1, MMP11, XDH, CP, COMP, GLB1L3, CXCL13, SULF1, LGSN, ABCC3, THBS2, KIF26B, GOLM1, KIAA0101, SIX1, ST6GALNAC1

Downregulated	MMRN1, KCNK3, RXFP1, RAMP3, SOCS2, FOXF1, FIBIN, KANK3, HBEGF, PCOLCE2, MFAP4, GNLY, IL33, AKAP12, ADAMTS1, S100A8, ACADL, RGCC, PLAC9, MS4A15, SGCG, HCAR3, ABI3BP, SCEL, AGTR1, LRRK2, ARHGAP6, LRRN3, CLDN5, TGFBR3, SFTPD, PIP5K1B, STXBP6, CFD, LDB2, ADAMTSL3, SCARA5, CALCRL, KCNT2, SNTN, ZBTB16, CXCL2, KIAA1462, ACKR1, OGN, MME, CDH5, GIMAP8, SCN7A, TAL1, ARHGEF26, LIN7A, ADH1B, DACH1, GNG11, CD300LG, KL, AFF3, PEBP4, GPIHBP1, C2orf40, KLF4, LYVE1, CHRDL1, MYZAP, ANKRD29, COL6A6, CDO1, LINC00968, CCBE1, CAV2, ZBED2, FHL1, AQP4, ROBO4, CXCR2, KAL1, PPBP, FILIP1, BCHE, SPOCK2, HBB, CA4, MAMDC2, SPTBN1, FGFBP2, INMT, FMO2, ADIRF, CCDC85A, TEK, FAM150B, TCF21, FAM107A, S100A12, CAV1, FOSB, STX11, MCEMP1, SERTM1, PDK4, SOX7, EDNRB, UPK3B, CPB2, ABCA8, IL6, IGSF10, IL1RL1, SDPR, BTNL9, EMCN, NCKAP5, MT1M, TNNC1, CLDN18, GKN2, SFTPC, CD36, SCGB1A1, SOSTDC1, FCN3, RTKN2, CLIC5, AGER, TMEM100, SLC6A4, FABP4, WIF1, GPM6A

**Table 2:** Gene Ontology of DEGs associated with nonsmoking woman lung cancer.

Category	Term	Count	*p* value	Gene name
GOTERM_BP_DIRECT	GO:0030574~collagen catabolic process	7	2.08*E* − 05	COL6A6, COL1A1, COL11A1, MMP12, MMP1, COL10A1, MMP11
GOTERM_BP_DIRECT	GO:0001937~negative regulation of endothelial cell proliferation	5	1.16*E* − 04	XDH, CAV2, CAV1, RGCC, SULF1
GOTERM_BP_DIRECT	GO:0030198~extracellular matrix organization	9	3.49*E* − 04	RXFP1, SPOCK2, FOXF1, COMP, COL1A1, COL11A1, ABI3BP, COL10A1, SPP1
GOTERM_BP_DIRECT	GO:0050729~positive regulation of inflammatory response	6	4.61*E* − 04	AGTR1, S100A8, IL1RL1, FABP4, IL33, S100A12
GOTERM_BP_DIRECT	GO:0031623~receptor internalization	5	5.47*E* − 04	RAMP3, CAV1, CD36, CXCR2, CALCRL
GOTERM_BP_DIRECT	GO:0001822~kidney development	6	9.76*E* − 04	TCF21, AGTR1, SIX1, SULF1, MME, ADAMTS1
GOTERM_CC_DIRECT	GO:0005576~extracellular region	41	1.02*E* − 09	EMCN, S100A8, CXCL2, IL33, MMRN1, MMP1, IGSF10, OGN, COL6A6, FCN3, BCHE, COMP, TEK, SFTPD, SFTPC, FAM150B, CFD, FIBIN, COL11A1, HBB, THBS2, SPP1, COL10A1, IL6, KL, AGER, MMP12, PLAC9, S100A12, MMP11, PCOLCE2, CHRDL1, CXCL14, PPBP, CXCL13, TGFBR3, HBEGF, WIF1, COL1A1, CP, MFAP4
GOTERM_CC_DIRECT	GO:0005578~proteinaceous extracellular matrix	17	1.87*E* − 09	CTHRC1, MAMDC2, ADAMTSL3, SPOCK2, IL1RL1, MMP1, MMP12, MMP11, OGN, COL6A6, COMP, CCBE1, SFTPD, TGFBR3, ADAMTS1, COL11A1, COL10A1
GOTERM_CC_DIRECT	GO:0005615~extracellular space	36	4.65*E* − 09	XDH, CTHRC1, S100A8, CXCL2, SPINK1, IL33, GREM1, SCGB1A1, ABI3BP, OGN, COMP, SOSTDC1, CCBE1, SFTPD, SFTPC, CFD, FGFBP2, GOLM1, SPP1, IL6, KL, GNLY, CST1, C2ORF40, GKN2, CD36, CXCL14, PPBP, CXCL13, SULF1, TGFBR3, HBEGF, COL1A1, CP, LRRK2, CPB2
GOTERM_CC_DIRECT	GO:0005581~collagen trimer	10	1.15*E* − 07	CTHRC1, CD36, COL6A6, FCN3, CCBE1, SFTPD, COL1A1, COL11A1, MMP1, COL10A1
GOTERM_CC_DIRECT	GO:0016323~basolateral plasma membrane	9	1.93*E* − 04	CAV1, PROM2, TEK, CD300LG, AQP4, CA4, CEACAM5, GPIHBP1, LIN7A
GOTERM_CC_DIRECT	GO:0045121~membrane raft	9	4.79*E* − 04	EDNRB, CAV2, CAV1, PROM2, CD36, SDPR, SLC6A4, SULF1, TEK
GOTERM_MF_DIRECT	GO:0008201~heparin binding	10	7.06*E* − 06	OGN, CXCL13, COMP, TGFBR3, HBEGF, ADAMTS1, THBS2, AGER, ABI3BP, PCOLCE2
GOTERM_MF_DIRECT	GO:0008009~chemokine activity	4	0.007634616	PPBP, CXCL14, CXCL13, CXCL2
GOTERM_MF_DIRECT	GO:0005518~collagen binding	4	0.013282483	COMP, CCBE1, ABI3BP, PCOLCE2
GOTERM_MF_DIRECT	GO:0004222~metalloendopeptidase activity	5	0.014206339	MME, ADAMTS1, MMP12, MMP1, MMP11
GOTERM_MF_DIRECT	GO:0017134~fibroblast growth factor binding	3	0.015208209	CXCL13, KL, TGFBR3
GOTERM_MF_DIRECT	GO:0005509~calcium ion binding	13	0.015389499	S100A8, TNNC1, SPOCK2, COMP, SULF1, CCBE1, MMRN1, THBS2, MMP1, CDH5, MMP12, S100A12, MMP11

**Table 3:** Significantly enriched KEGG pathway of differentially expressed genes (DEGs).

Term	Count	*p* value	Gene name
hsa05144:malaria	6	1.27*E* − 04	IL6, CD36, COMP, ACKR1, THBS2, HBB
hsa04512:ECM-receptor interaction	7	2.36*E* − 04	CD36, COL6A6, COMP, COL1A1, COL11A1, THBS2, SPP1
hsa04974:protein digestion and absorption	6	0.001924368	COL6A6, MME, COL1A1, CPB2, COL11A1, COL10A1
hsa04510:focal adhesion	8	0.004729076	CAV2, CAV1, COL6A6, COMP, COL1A1, COL11A1, THBS2, SPP1
hsa04151:PI3K-Akt signaling pathway	9	0.023099212	IL6, COL6A6, COMP, TEK, GNG11, COL1A1, COL11A1, THBS2, SPP1
hsa03320:PPAR signaling pathway	4	0.030418433	CD36, FABP4, ACADL, MMP1
hsa04062:chemokine signaling pathway	6	0.04032297	PPBP, CXCL14, CXCL13, CXCL2, CXCR2, GNG11

**Table 4:** Gene Ontology of 5 hub genes through Metascape.

Gene	Gene full name	Biological process (GO)	GO term
FOSB	Proto-oncogene	Response to corticosterone Response to isoquinoline alkaloid Response to morphine	GO:0051412 GO:0014072 GO:0043278
IL33	Interleukin 33	Regulation of bone trabecula formation Negative regulation of bone trabecula formation Negative regulation of osteoclast proliferation	GO:1900154 GO:1900155 GO:0090291
GREM1	Gremlin 1 DAN family BMP antagonist	Regulation of cellular defense response Positive regulation of cellular defense response Negative regulation of macrophage proliferation	GO:0010185 GO:0010186 GO:0120042
MMP11	Matrix metallopeptidase 11	Basement membrane organization Collagen catabolic process Negative regulation of fat cell differentiation	GO:0071711 GO:0030574 GO:0045599
SPP1	Secreted phosphoprotein 1	Collateral sprouting of intact axon in response to injury Regulation of collateral sprouting of intact axon in response to injury Negative regulation of collateral sprouting of intact axon in response to injury	GO:0048673 GO:0048683 GO:0048685

## Data Availability

The data used to support the findings of this study are included within the article.

## Abbreviations

BP: Biological process
CC: Cellular component
DEGs: Differentially expressed genes
ECM: Extracellular matrix
GEO: Gene Expression Omnibus
GO: Gene Ontology
GREM1: Gremlin 1
KEGG: Kyoto Encyclopedia of Genes and Genomes
LC: Lung cancer
MMP11: Matrix metallopeptidase 11
IL33: Interleukin 33
PPI: Protein-protein interaction
FOSB: Proto-oncogene
SPP1: Secreted phosphoprotein 1
VEGF: Vascular endothelial growth factor.
